# A new Purkinje cell antibody (anti-Ca) associated with subacute cerebellar ataxia: immunological characterization

**DOI:** 10.1186/1742-2094-7-21

**Published:** 2010-03-12

**Authors:** Sven Jarius, Klaus P Wandinger, Sigrun Horn, Heike Heuer, Brigitte Wildemann

**Affiliations:** 1 Division of Molecular Neuroimmunology, Department of Neurology, University of Heidelberg, Heidelberg, Germany; 2 Institute for Neuroimmunology and Clinical MS Research, Center for Molecular Neurobiology Hamburg (ZMNH), University Medical Center Eppendorf, Hamburg, Germany; 3 Institute for Experimental Immunology, affiliated to Euroimmun, Luebeck, Germany; 4 Leibniz Institute for Age Research/Fritz Lipmann Institute, Jena, Germany

## Abstract

We report on a newly discovered serum and cerebrospinal fluid (CSF) reactivity to Purkinje cells (PCs) associated with subacute inflammatory cerebellar ataxia. The patient, a previously healthy 33-year-old lady, presented with severe limb and gait ataxia, dysarthria, and diplopia two weeks after she had recovered from a common cold. Immunohistochemical studies on mouse, rat, and monkey brain sections revealed binding of a high-titer (up to 1:10,000) IgG antibody to the cerebellar molecular layer, Purkinje cell (PC) layer, and white matter. The antibody is highly specific for PCs and binds to the cytoplasm as well as to the inner side of the membrane of PC somata, dendrites and axons. It is produced by B cell clones within the CNS, belongs to the IgG1 subclass, and activates complement in vitro. Western blotting of primate cerebellum extract revealed binding of CSF and serum IgG to an 80-97 kDa protein. Extensive control studies were performed to rule out a broad panel of previously described paraneoplastic and non-paraneoplastic antibodies known to be associated with cerebellar ataxia. Screening of >9000 human full length proteins by means of a protein array and additional confirmatory experiments revealed Rho GTPase activating protein 26 (ARHGAP26, GRAF, oligophrenin-1-like protein) as the target antigen. Preadsorption of the patient's serum with human ARHGAP26 but not preadsorption with other proteins resulted in complete loss of PC staining. Our findings suggest a role of autoimmunity against ARHGAP26 in the pathogenesis of subacute inflammatory cerebellar ataxia, and extend the panel of diagnostic markers for this devastating disease.

## Background

Autoimmune cerebellar ataxia (ACA) is an etiologically and pathologically heterogeneous syndrome. Besides multiple sclerosis (MS), paraneoplastic neurological disorders (PND) are the most common cause of ACA[1,2]. Many cases of paraneoplastic ACA are associated with serum or CSF antibodies to neuronal and/or glial antigens such as anti-Hu[3], anti-Yo[4], anti-CV2/CRMP5[5,6], anti-Tr[7], anti-Zic4[8], anti-protein kinase C gamma (PKCγ)[9], anti-mGluR1[10,11], anti-PCA2[12], anti-ANNA3[13], or antibodies to voltage gated calcium channels (VGCC)[14]. In patients with non-paraneoplastic ACA, antibodies to glutamate decarboxylase[15,16], tissue transglutaminase[17], glutamate receptor δ2 (GluRδ2)[18,19], and Homer-3[20] have been described.

Here we report a newly discovered autoantibody to Purkinje cells in a patient with subacute cerebellar ataxia but no tumor. This antibody binds specifically to the inner membrane and cytoplasm of PC somata, dendrites and axons. It is produced intrathecally, belongs to the IgG1 subclass and activates complement in vitro. Probing of a protein microarray with the patient's serum and additional confirmatory experiments identified the Rho GTPase activating protein 26 (ARHGAP26) as the target antigen.

## Case history

**Figure 1:**
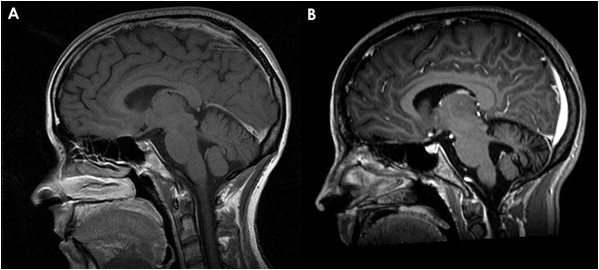
**Magnetic resonance imaging of the cerebellum demonstrating marked cerebellar atrophy over the course of disease but no contrast enhancement**. (A) T1-weighted sagittal sequence; image obtained at month 3 after onset. (B) Gadolinium-enhanced sagittal T1-weighted sequence; image obtained at month 17. Note the widening of the cerebellar sulci and the fourth ventricle.

A 33-year-old Caucasian lady was admitted to our hospital with a five-day history of diplopia, slurred speech, and gait instability. Two weeks before onset of symptoms she had recovered from a common cold. Neurologic assessment revealed horizontal nystagmus, dysarthria, limb ataxia predominantly affecting the right upper extremity, and severe gait ataxia. Cranial and spinal magnetic resonance imaging (MRI), ultrasound imaging of cerebral vessels, visual, acoustic, and somatosensory evoked potentials as well as nociceptive blink and trigeminal inhibition reflexes were normal. CSF analysis disclosed 44 lymphocytes/μl with few plasma cells, mild blood/CSF barrier dysfunction, increased CSF ratios of IgG and IgM, and CSF-restricted oligoclonal bands (OCB). Serology and/or PCR were negative for HSV1, HSV2, VZV, EBV, HHV6, enterovirus, arbovirus, HBV, HCV, HIV, borrelia, treponema. Routine blood analysis (including CRP, blood sedimentation rate, and chromogranin A) was unremarkable except for a slightly elevated titer of serum antinuclear antibodies (1:320). No antibodies to extractable nuclear antigens were detectable. Testing for anti-neutrophil cytoplasmic antibodies was negative. The presumptive diagnosis was postinfectious cerebellitis, and the patient was treated with 3 × 1000 mg methylprednisolone (MP) intravenously followed by oral therapy over three weeks at an initial dose of 60 mg. The corticosteroid therapy resulted in marked neurological improvement, but was associated with restlessness, sleeplessness, depressed mood, and suicidal thoughts. After tapering corticosteroids to 12.5 mg MP per day the patient experienced worsening of symptoms together with an exaggerated startle response. Clinical examination demonstrated tetra-ataxia, severe gait ataxia, oscillopsia, and marked dysarthria. Moreover, a brisk head retraction reflex was noted. The CSF had a normal cell count and persistent intrathecal IgG synthesis. Routine laboratory tests, a broad panel of tumor markers, thyroid hormones, anti-thyroid antibodies (anti-thyroglobulin, anti-thyroid peroxidase, anti-TSH receptor), anti-cardiolipin, and common anti-neuronal antibodies (anti-Hu, -Yo, -Ri, -Ma/Ta, -CV2/CRMP5, -GAD, -amphiphysin) were normal or negative. Whole body positron emission tomography (PET) scans three, six and 13 months after onset, repeat computed tomography (CT) of the chest and abdomen, abdominal ultrasound and gynecologic examinations did not show evidence of cancer. Repeat brain MRI was negative two months after onset, but disclosed cerebellar atrophy at month 4 (Figure 1). An FDG-PET examination at month 6 revealed a relative hypometabolism of the right cerebellar hemisphere. The exaggerated startle response was stabilized by lorazepam (0.5 mg/d). A further course of intravenous MP pulse therapy (5 × 500 mg) followed by treatment with 3 × 30 g intravenous immunoglobulins (IVIG) did not promote improvement of neurologic deficits significantly. The cerebellar syndrome finally stabilized and improved following plasma exchange (two courses of 4 and 3 treatments) and immunoadsorption (two courses of 5 and 6 treatments). At last evaluation, 16 months after onset, the patient presented with cerebellar signs and hyperekplexia without further progression and without evidence of an underlying malignancy. The study was approved by the institutional Review Board of the Medical Faculty of the University of Heidelberg and the patient gave written informed consent.

## Immunological evaluation

### Methods

#### Immunohistochemistry (IHC)

IHC was performed on cryosections of adult mouse cerebellum, cerebrum, and brain stem tissue (Euroimmun, Luebeck, Germany), on cryosections of adult rhesus monkey cerebellum, brain stem, cerebrum, hippocampus, hypothalamus, peripheral nerve, and intestine tissue (Euroimmun), and on cryosections of adult rat cerebellum and brain stem (Zyagen, San Diego, CA). Mouse and monkey tissue was provided unfixed as snap frozen sections (4-6 μm) and was fixed with 10% formalin in phosphate buffered saline (PBS) for 4 min before testing. For production of rat sections, animals had been perfused with 4% paraformaldehyde (PFA), the brains removed, immersed to the same fixative up to 12 hours, cryoprotected with sucrose, frozen in OCT, and finally sectioned at a thickness of 4-10 μm. For some experiments, 0.125% triton-X or 1% 3- [(3-cholamidopropyl)dimethylammonio]-1-propanesulfonate (CHAPS) in PBS were applied to the sections for 4 min. Sections were then washed in PBS, blocked with 10% goat serum or with 10% donkey serum, with respect to the secondary antibodies used, and, after three washes in chilled PBS, incubated with patient serum for 1 hour or with various commercial antibodies for three hours at room temperature (RT) or overnight at 4°C. Binding of human IgG, IgA and IgM to CNS tissue was detected by use of polyclonal goat anti-human IgG antibodies conjugated to fluorescein isothiocyanate (FITC) (Euroimmun), Alexa Fluor^® ^(AF) 488 (Invitrogen, Karlsruhe, Germany) or AF568 (Invitrogen), polyclonal donkey anti-human IgG antibodies labeled with Rhodamin Red-X (Dianova, Hamburg, Germany), and polyclonal goat anti-human IgM and anti-human IgA antibodies conjugated to FITC (Euroimmun), respectively. Binding of the following commercial antibodies was detected using goat anti-rabbit IgG AF568 (1:200-1:300; Invitrogen), goat anti-mouse IgG AF568 (1:100-1:400; Invitrogen), donkey anti-chicken IgG Rhodamin-Red × (1:100-1:200; Dianova), or donkey anti-goat IgG AF488 (1:200-1:400) as secondary antibodies depending on the primary antibodies employed and on further secondary antibodies used in double labeling experiments: goat anti-Homer3 (1:50; Santa Cruz, Heidelberg, Germany); rabbit anti-protein kinase C gamma (PKCγ) (1:50; Santa Cruz); mouse anti-metabotropic glutamate receptor 1α (mGluR1α) (1:200-1:300; BD Pharmingen, Heidelberg, Germany); rabbit anti-glutamate receptor delta 2 (GluRδ2) (1:25-1:50; Santa Cruz); mouse anti-glutamate receptor 3 (GluR3, clone 3B3) (1:25-1:500; Millipore, Schwalbach, Germany); rabbit anti-inositol-triphosphate receptor type I (IP3RI) (1:200; Dianova); chicken anti-glial fibrillary acidic protein (GFAP) (1:1000; Encor Biotechnology, Gainesville, FL); rabbit anti-aquaporin4 (AQP4) (1:200; Sigma Aldrich, Taufkirchen, Germany); mouse anti-calbindin-D (1:50; Swant, Bellinzona, Switzerland); goat anti-parvalbumin (1:50; Swant); and anti-Rho GTPase-activating protein 26 (ARHGAP26) (1:75; Santa Cruz). For selected experiments, the patient's CSF was incubated with 3 μg of the following recombinant human proteins for 3 h at RT prior to testing: ARHGAP26 (Abnova, Taipei, Taiwan); IP3RI (Santa Cruz); and AQP4 (Abcam); the sera were then centrifuged at 11,2000 rpm for 10 min and the supernatants incubated with cerebellum sections as described above. Sections were then mounted using glycerol standard immunofluorescence mounting medium containing 4',6-diamidino-2-phenylindole (DAPI) (1:1000) (Euroimmun) or ProLong Gold antifade reagent (Invitrogen). Slides were analyzed on a Nikon 90i upright fluorescence microscope and a Nikon A1 confocal microscope (Nikon Imaging Center, University of Heidelberg, Heidelberg, Germany).

#### IgG subclass analysis

For evaluation of IgG subclasses, serum and CSF samples were tested by IHC on mouse cerebellum sections as described above, with the following modifications applied: unconjugated sheep anti-human IgG antibodies specific for IgG subclasses 1 to 4 (Binding site, Germany) were substituted for the FITC-labeled goat anti-human IgG antibody, and AF568 labeled donkey anti-sheep IgG (Invitrogen; absorbed against human IgG) was used to detect the subclass specific antibodies.

#### Complement assay

For evaluation of complement activation, mouse cerebellum sections were incubated with heat inactivated serum samples (60 min at 56°C) from our patient or controls 1:5 dilution for 60 minutes at 37°C. After three washes in chilled PBS, pooled fresh frozen serum from three healthy donors was applied as a source of complement at 1:5 dilution for 45 minutes at 37°C, followed by fixation with 10% formalin for 15 min on ice and incubation with CHAPS for 1 min. Sections were then blocked with 10% heat inactivated goat serum for 60 min and subsequently incubated with a polyclonal rabbit anti-human C3c/C3b antibody at a dilution of 1:250 for 45 minutes at 4°C (Dako, Hamburg, Germany), or a rabbit isotype control (ready for use; Zymed Laboratories, San Francisco, California) on ice. Binding of the C3c/C3b specific antibody was visualized using a goat anti-rabbit IgG AF568 antibody (1:300; 45 min; Invitrogen). Sections were washed in chilled PBS between incubations and prior to mounting.

#### Western blot

Serum and CSF samples were tested for the presence of antibodies to cerebellar antigens by use of a commercially available western blot assay (Euroimmun, Germany). Briefly, ready-made nitrocellulose membranes containing rhesus monkey full cerebellum extract were blocked with a ready-made blocking buffer (Euroimmun) for 15 min, incubated overnight at 4°C with diluted serum (1:25) or CSF (1:4) from the patient or healthy controls, washed three times in diluted blocking buffer, and finally visualized using an alkaline phosphatase labeled anti-human IgG antibody as conjugate and BCIP (5-bromo-4-chloro-3'-indolyphosphate p-toluidine salt) and NBT (nitro-blue tetrazolium chloride) as chromogenes. The reaction of BCIP/NBT to 5-5'-dibromo-4,4'-dichloro-indigo and NBT-formazan was stopped after 10 min by application of ice cold ddH_2_O. Additional membrane stripes originating from the same blot were incubated with goat anti-Homer3 (1:100), rabbit anti-PKCγ (1:75), mouse anti-mGluR1α (1:200), rabbit anti-GluRδ2 (1:75), rabbit anti-IP3RI (1:500), rabbit anti-coilin (Santa Cruz) (1:100), and rabbit anti-ARHGAP26 (Santa Cruz), respectively. Binding of these antibodies to their respective antigens was visualized using the Odyssee^® ^Infrared Imaging System after application of goat anti-rabbit IgG labeled with Infrared dye (IRdye) 800 (Rockland Immunochemicals, Gilbertsville, PA) or goat anti-mouse AF688 (Invitrogen) as secondary antibodies (1:5000). A batch-specific evaluation matrix provided by the manufacturer was used to identify molecular weight (MW) ranges for proteins detected by the patient's serum and CSF or either of the commercial antibodies used. In addition, bands detected with well established paraneoplastic antibodies (Ri, 80kDa; Yo, 62 and 34 kDa; Ri, 55 kDa; Hu, 38kDa) were used to further define MW ranges.

#### Intrathecal antibody synthesis

Oligoclonal IgG bands were assessed by standard methods employing isoelectric focusing of serum and CSF at equal concentrations and subsequent detection of IgG by immunoblotting. Quantitative expressions of intrathecal antibody synthesis were based on calculation of the CSF/serum ratios of Purkinje cell specific IgG antibodies and total IgG (QIgG [spec] = IgGspec [CSF]/IgGspec [serum], and QIgG [total] = IgGtotal [CSF]/IgGtotal [serum]) [21]. Antibody titers were determined semi-quantitatively by indirect immunofluorescence on mouse cerebellum sections as described above. Total IgG and total albumin concentrations in serum and CSF were determined nephelometrically (BN ProSpec, Dade Behring, Germany). The intrathecal synthesis of anti-Purkinje cell antibodies was detected by calculation of the corresponding antibody index (AI): AI = QIgG [spec]/QIgG [total], if QIgG [total] < Qlim, and AI = QIgG [spec]/Qlim, if QIgG [total] > Qlim[21]. The upper reference range of QIgG [total], Qlim, was calculated according to Reiber's formula to correct for possible underestimation of intrathecal specific synthesis due to possible blood-CSF barrier disturbance[21]. AI values >4 were considered to be indicative of intrathecal anti-Purkinje cell IgG production[22].

#### Testing for co-existing autoantibodies

Commercially available immunoblots were used to test for antibodies to Hu, Yo, Ri, amphiphysin, glutamate decarboxylase (GAD), CV2/CRMP5, Ma2/PNMA2, GM1, GM2, GM3, GD1a, GD1b, GT1b, GQ1b, and AMA-M2 according to the manufacturer's instructions (Euroimmun). ANAs were assessed by immunocytochemistry on HEp2 cells and further characterized by commercial immunoblots for the detection of antibodies to nRNP/Sm, Sm, and SS-A, Ro-52, SS-B, Scl-70, PM-Scl, Jo-1, PCNA, double stranded DNA (dsDNA), centromer protein B (CEPB), nucleosomes, histones, and ribosomale P proteins (Euroimmun), and by double-staining of tissue sections with a rabbit polyclonal antibody to p80-coilin (Santa Cruz).

#### Protein array

A commercially available human protein microarray (Protoarray v5.0; Invitrogen) spotted with >9000 human full length-proteins purified from a baculovirus-based expression system was probed with the patient's serum according to the manufacturer's instructions. Briefly, the array was incubated with blocking buffer containing 50 nM HEPES, pH 7.5, 200 nM NaCl, 0.08% triton X-100, 25% glycerol, 20 nm reduced glutathione, 1 nM DTT, and a commercial synthetic block (Invitrogen) for 1 h at 4°C. After washing, the array was incubated with the patient's serum at a 1:1500 dilution in washing buffer containing PBS-0.1%Tween and synthetic block. After more washing steps, a goat anti-human IgG detection antibody labeled with AF647 (final concentration, 1 μg/μl) (Invitrogen) was applied for 90 min at 4°C. A Genepix™ 4000 B microarray scanner and corresponding application software (Genepix, Sunnyvale, CA) was used to scan the slide, and Protoarray v5.0 Prospector software (Invitrogen) to analyze the raw data.

#### Dot blot assay

Protran BA79 nitrocellulose membranes (0.1 μm) (Whatman) were spotted with increasing dilutions (1:2, 1:4, 1:8, 1:16, 1:32) of a 0.14 μg/μl solution of human full length ARHGAP26 (10 μl/spot) in 0.1% bovine serum albumin (BSA). After drying, membranes were blocked with 5% bovine serum albumin (BSA) in Tris-buffered saline (TBS) for 1 h at RT, washed three times in TBS with 0.05% Tween (TBS-T), and finally incubated with a 1:20 or 1:200 dilution of the patient's serum in 0.1% BSA/TBS-T for 1 h at RT. A donkey anti-human IgG antibody labeled with IRdye 700DX (Rockland) was used to detect bound IgG. Stripes were finally washed in TBS and analysed using an Odyssey™ fluorescence scanner (Licor, Lincoln, NE) and Odyssey™ 2.0.40 application software (Licor). As controls, serum samples from three healthy donors were tested in the same run.

#### Mixed cerebellar cultures

Mixed cerebellar cultures were prepared as published previously[23,24]. Briefly, newborn NMRI mice were killed by decapitation. Cerebella were dissected in PBS, and meninges were removed. Tissues were treated with trypsin (1% in PBS; Worthington, Freehold, NJ) for 3 min at room temperature (RT). After replacing trypsin with DNase (0.05% in BME; Worthington), cerebella were triturated successively with three fire-polished Pasteur pipettes of decreasing bore sizes. Cells were centrifuged and resuspended in PBS with DNase, and the cell slurry was passed through a 40 μM nylon mesh filter. Cells were resuspended in serum containing medium, incubated in uncoated Petri dishes for 30 min at 37°C to remove fibroblast contamination, and finally plated on coverslips coated with 500 μg/ml poly-D-lysine. Plated cells were allowed to attach overnight, and then the medium was changed to complete serum-free medium composed of BME, BSA (10 mg/ml; A-8806, Sigma), glutamax (10 mM), glucose (32 mM), 1 nM triiodothyronine (Sigma-Aldrich), penicillin-streptomycin (29 U/ml each, Invitrogen) and Sigma I-1884 supplement. Thereafter, medium was replaced every 3-4 d during culture period of 14 days.

#### Immunocytochemistry

Purkinje cell cultures were fixed with 4% paraformaldehyde and 0.05% triton-X at RT for 30 min and immunostained with the patient's serum (1:1000) or a mouse monoclonal antibody against calbindin D28k (1:500; Sigma-Aldrich) followed by incubation with an AF488- or AF555-labeled goat anti-mouse or goat anti-human IgG antibody (1:1000; Invitrogen). Cells were then photographed with an AxioCam digital camera (Zeiss, Germany) on a Zeiss Axio Imager microscope (63× objective).

## Results

### Detection of a Purkinje cell-specific antibody in the CSF

**Figure 2:**
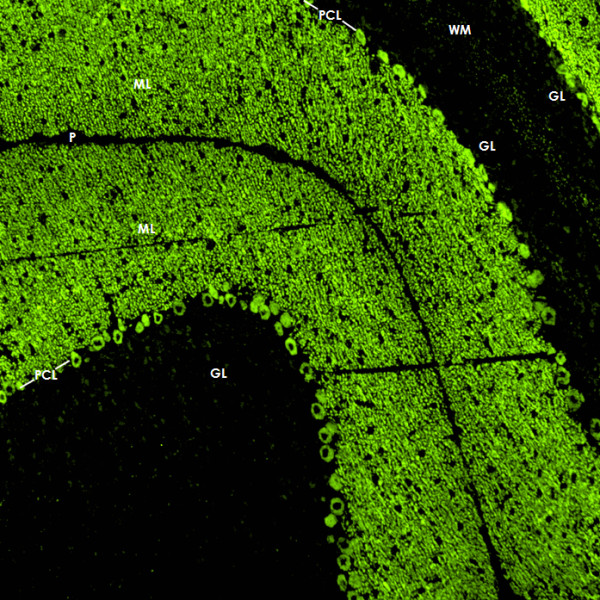
**Binding of CSF IgG to the molecular layer (ML), the Purkinje cell layer (PCL) and the white matter (WM) on a mouse cerebellum tissue section**. An AlexaFluor^® ^488 labeled goat anti-human IgG antibody was used to visualize bound patient IgG. GL = granular layer, P = pia mater.

**Figure 3:**
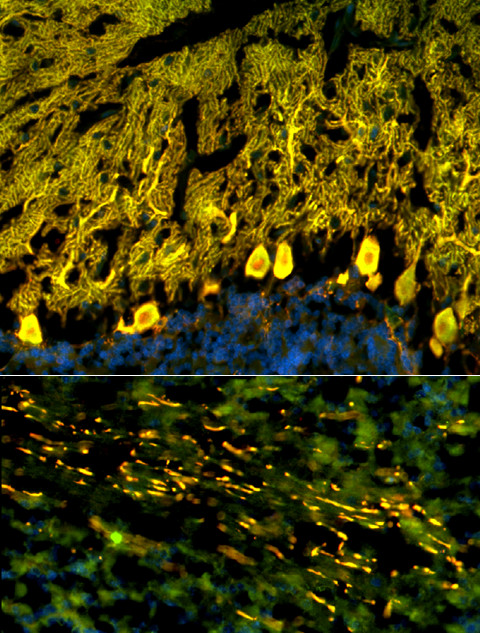
**Double labeling with an antibody to calbindin, a specific marker of Purkinje cells (PCs), proved that the cellular structures targeted by the patient's CSF IgG correspond to PC somata, PC dendrites (upper panel), and PC axons (lower panel)**. Anti-calbindin reactivity is depicted in red (AlexaFluor^® ^568); the patient's antibody in green (AlexaFluor^® ^488); and yellow color indicates overlay of the two antibodies. Nuclei are shown in blue (DAPI). Note that the patient's antibody spared the PC nucleus.

**Figure 4:**
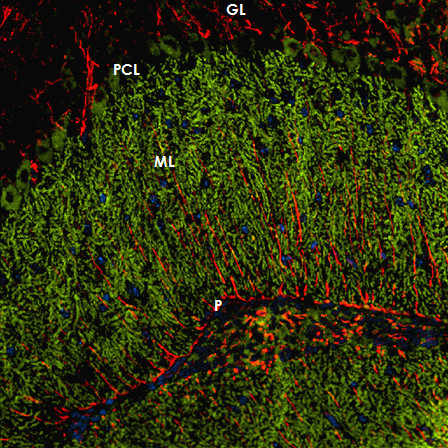
**The patient's antibody bound selectively to Purkinje cells but not to astrocytes such as the Bergman glial cells (BGC) as demonstrated by double staining with an antibody to anti-glial fibrillary acidic protein (GFAP)**. The anti-GFAP antibody, staining astrocytes in the granular layer (GL) as well as the processes of the BGCs in the molecular layer (ML) and along the pia mater (P), is depicted in red (AF568); and the patient's antibody is labeled in green (AF488); yellow color would indicate overlay of the two antibodies, but is absent. Nuclei are shown in blue.

**Figure 5:**
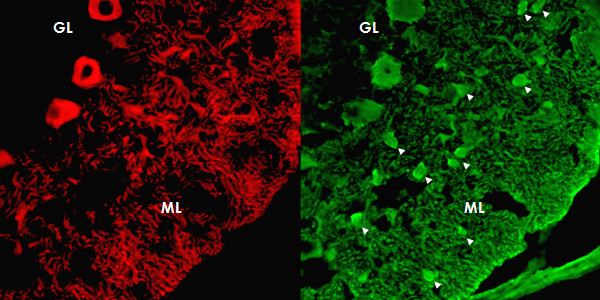
**Double staining of the cerebellar cortex with an antibody to parvalbumin (green; AF488), a general marker of cerebellar neurons, demonstrates that the patient's antibody (red; AF568) binds neither to interneurons (*arrow heads*) in the molecular layer (ML) nor to granular cells in the granular layer**.

Immunohistochemistry on formalin-fixed frozen adult mouse, monkey and rat tissue sections demonstrated strong reactivity of CSF and serum to structures in the molecular layer (ML), the Purkinje cell layer (PCL), and the white matter (WM) of the cerebellum (Figure 2). More detailed analysis at higher magnification revealed binding of IgG to somata, dendritic trunks, dendritic branches, and possibly dendritic spines of Purkinje cells (PCs). In addition, axons in the WM were stained by the patient's CSF and serum. Specific binding to Purkinje cells (PC) was indicated by morphology and confirmed by double labeling of PCs with the patient antibody and an anti-calbindin antibody (Figure 3). Double labeling with anti-GFAP and anti-AQP4 revealed no binding of the patient antibody to astrocytes in the WM and the granular layer (GL), or to Bergman glial cells in the PCL and ML (Figure 4). Binding to the soma of interneurons such as stellate cells, basket cells and Golgi cells or to granular cells was excluded by double labeling with parvalbumin and the patient's CSF and serum (Figure 5). Testing of formalin-fixed tissue sections of cerebral cortex and white matter, brain stem, hippocampus, hypothalamus, peripheral nerve, and plexus myentericus under identical conditions was negative except for single cell somata in the brain stem and cerebral white matter (not shown). We decided to refer to the specific staining pattern described here as to anti-Ca throughout the manuscript, following a widely accepted convention to name newly described antibodies with reference to the index patient.

### The antibody is produced intrathecally

A titer of 1:2000 was found with the first CSF sample obtained (taken five days after onset of symptoms). A paired serum sample taken at the same time yielded a titer of 1:6000. This corresponded to an anti-Ca index of 68 when related to CSF and serum albumin (Qlim; see method section) in the same samples (Table 1), indicating autochthonous production of the antibody within the CSF. Intrathecal production of total IgG was further indicated by the presence of CSF-restricted oligoclonal bands and an increase of both Reiber's IgG ratio (Q_IgG _= 6.44; Q_Alb _= 6.66)[21] and Link's IgG index (0.97). Sustained, though markedly reduced, intrathecal production of both total IgG and the PC-specific antibody was demonstrated in a paired follow-up sample obtained two months after commencement of corticosteroid therapy (Table 1).

**Table 1:** Laboratory findings, treatment, and treatment response over the course of disease

Months from onset	Cells/μl CSF	Total protein mg/dl	IgG anti-Ca titers (IHC)		QIgG anti-Ca	IgG total mg/dl		QIgG	IgM total mg/dl		QIgM	Albumin total mg/dl		QAlb	Qlim	AI anti-Ca	OCBs CSF/serum
			**CSF**	**Serum**		**CSF**	**Serum**		**CSF**	**Serum**		**CSF**	**Serum**				

0	44 ↑	48 ↑	1:2000	1:6000	333.3	7.66	1190	6.44	0.567	267	2.12	25.1	3770	6.66	4.89	68.2	Pos/neg

Interventions:	3 × 1000 mg methylprednisolone (MP) intravenously followed by oral therapy over 3 weeks at an initial dose of 60 mg MP, resulting in marked neurological improvement. After tapering corticosteroids to 12.5 mg MP per day the patient experienced worsening of symptoms together with an exaggerated startle response (month 2).																

2	1	31	N.d.	N.d.	N.d.	3	813	3.69	0.086	296	0.29	10.4	4410	2.36	1.46	N.d.	N.d.

Interventions:	5 × 500 mg methylprednisolone (MP) intravenously (month 3) and 5 × 500 mg methylprednisolone (MP) intravenously + intravenous immunoglobulins (month 4), followed by only slight and transient neurological improvement. Oral therapy with MP at an initial dose of 60 mg MP.																

5	2	25	1:200	1:7000	28.6	1.9	927	2.05	0.065	251	0.26	14.7	3950	3.72	2.44	11.7	Pos/neg

Interventions:	Plasma exchange at month 6 (after progressive neurological deterioration), followed by clinical stabilization and moderate improvement.																

AI = antibody index (see method section for details); CSF = cerebrospinal fluid; IHC = immunohistochemistry; QIgG = IgG CSF/serum ratio; QIgM = IgM CSF/serum ratio; QAlb = albumin CSF/serum ratio; Qlim = upper reference range for IgG, albumin-dependent (see method section for details); OCB = oligoclonal bands.

### The antibody belongs to the IgG1 subclass and activates complement in vitro

**Figure 6:**
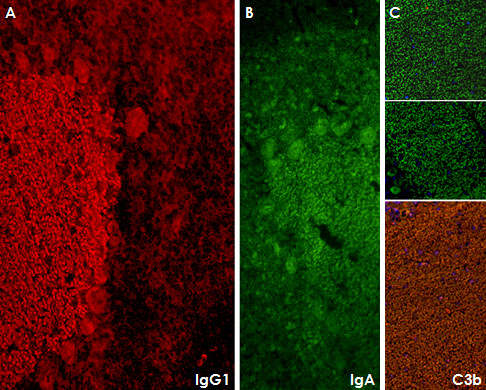
**Subclass analysis revealed that the antibody belongs mainly to the IgG1 subclass (panel A)**. In addition, antibodies of the IgA subclass were detectable (panel B). In line with the presence of IgG1 antibodies, fixing of complement C3b was found (panel C; the two upper images show negative control experiments with an isotype control and without complement, respectively). Only very weak IgG3 reactivity and no IgG2, IgG4, or IgM antibodies were detectable in serum or CSF (not shown).

The antibody belonged mainly to the IgG1 subclass both in the CSF and in the serum with only very weak staining for IgG3 and IgA (Figure 6). In line with this finding, use of fresh human serum as complement source resulted in C3b deposition with a distribution highly similar to that of the IgG1 antibodies (Figure 6), indicating complement activation by the patient's antibody. No IgM, IgG2, or IgG4 antibodies to PCs were detectable in CSF and serum.

### The antibody recognizes a 80-97 kDa protein

**Figure 7:**
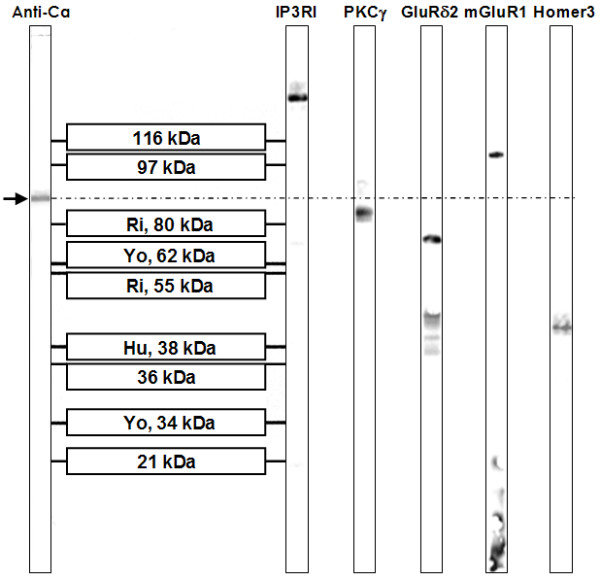
**A commercial western blot of primate cerebellum extract revealed binding of CSF IgG to a 80-97 kDa band, which does not correspond to bands found with antibodies to inositol-triphosphate receptor type I (IP3RI), protein kinase C gamma (PKCγ), glutamate receptor delta 2 (GluRδ2), metabotropic glutamate receptor 1α (mGluR1α), or Homer3**.

A distinct single band between 80 kDa and 97 kDa was detected when the initial CSF sample taken five days after onset was tested using a commercial western blot of primate total cerebellum extract. The same single band was present in a follow-up CSF sample obtained two months after onset of symptoms (Figure 7). Serum analysis demonstrated a corresponding band at the same position.

### The antibody targets an antigen sited at the plasma membrane and in the cytoplasm of Purkinje cells

**Figure 8:**
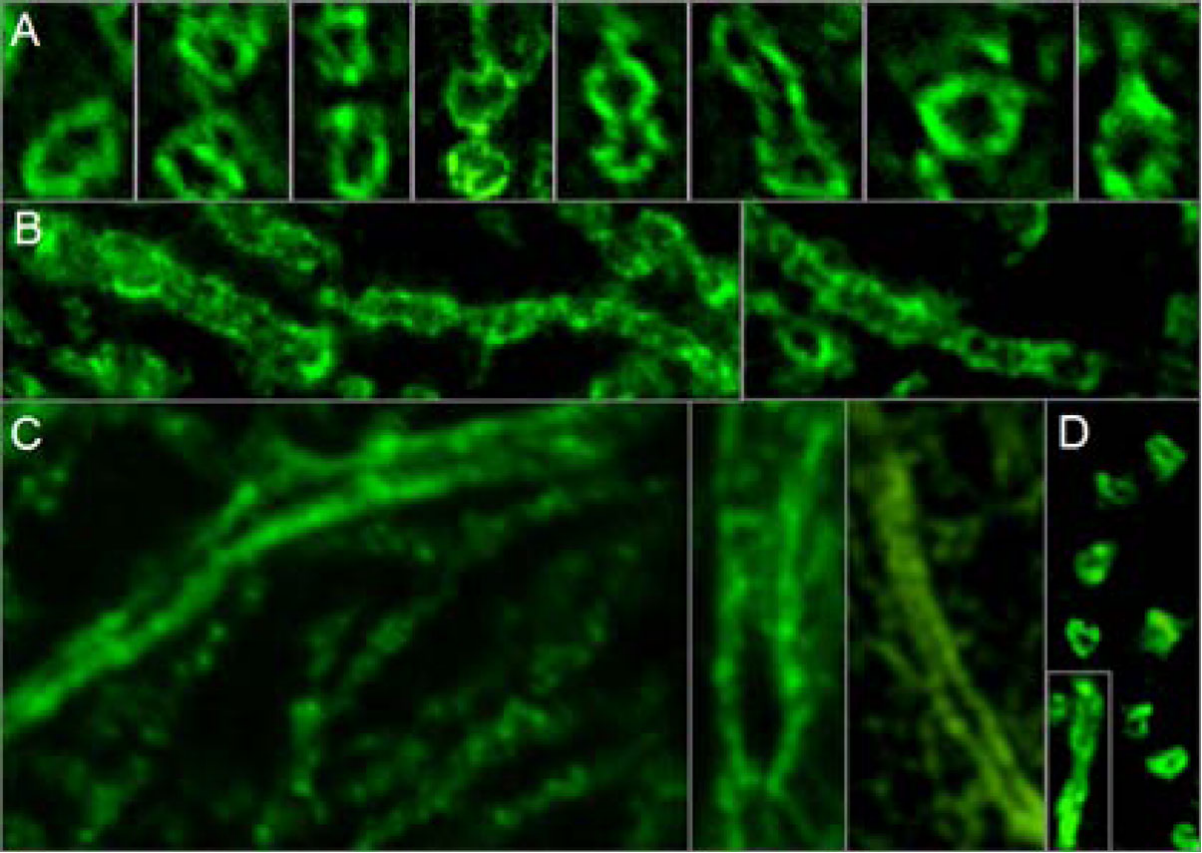
**High magnification revealed binding of IgG from the patient's CSF to membranes of PC dendrites and axons**. Panel A shows transverse sections and panel B depicts longitudinal sections of distal PC dendrites (mouse tissue). Panel C shows three longitudinal sections of proximal PC dendrites (monkey tissue). Panel D consists of a composite of transverse and longitudinal sections of monkey PC axons. A goat anti-human IgG antibody labeled with AF488 was used to visualize bound IgG.

**Figure 9:**
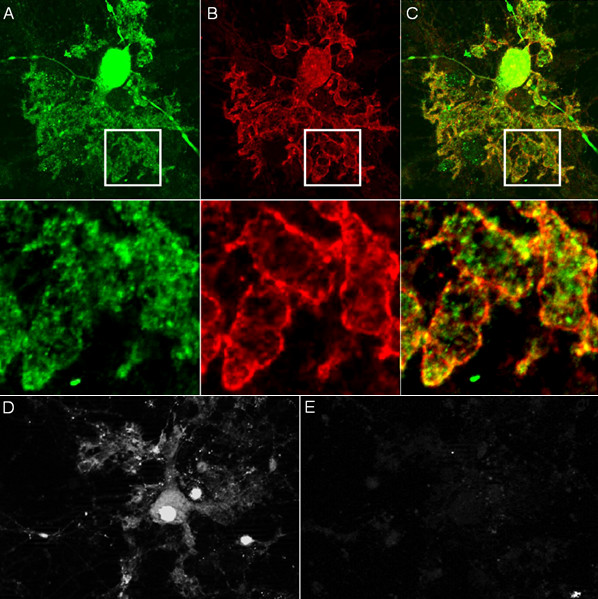
**Double staining of cultured Purkinje cells (PC) with the patient's serum revealed binding of IgG to PC somata and to membranes of PC dendrites and axons**. Calbindin is depicted in green (A; AF488); the patient's antibody in red (B; AF555); yellow color indicates overlay of the two antibodies (C). Binding was only observed after fixation with 4% paraformaldehyde and 0.05% triton-X (A-C), but not on living cells (D-E), suggesting an intracellular localization of the target antigen. Panel D shows fluorescence of a GFP-transfected living (i.e. unfixed) PC; Panel E demonstrates lack of binding of the patient' serum to the same cell.

**Figure 10:**
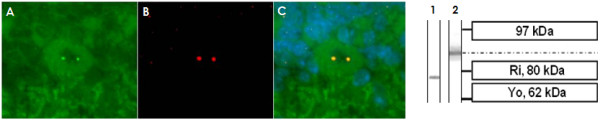
**Double staining of cerebellum tissue sections with a commercial antibody to p80-coilin identifies the dotted nuclear staining seen with the broad majority of cerebellar neurons following incubation with the patient's serum (but not CSF) as anti-coilin immunoreactivity**. The patient's antibody is depicted in green (A; AF488); the commercial coilin antibody in red (B; AF568); yellow color indicates overlay of the two antibodies (C). The p80-coilin antibody recognized a ~80 kDa protein band (lane 1) in a western blot of primate cerebellum tissue, which was not identical to the 80-97 kDa band found with the patient's serum and CSF (lane 2).

Immunohistochemistry demonstrated binding of IgG from the patient's CSF to the cytoplasm of PC somata (Figure 2) as well as to the plasma membrane of PC dendrites and PC axons (Figure 8 and 9). No nuclear staining (except for coilin in the serum but not CSF; see below) was detectable (Figure 10). However, permeabilization was found to be a prerequisite of binding to cultured PCs, indicating that the antigen is not a trans-membrane protein but rather located at the inner side of the membrane (Figure 9). In contrast, fixation and permeabilization was not required for membrane binding in the IHC assay, most likely due to most PCs having being transected during preparation of the 4-7 μm cryosections. This is important for potential future diagnostic applications, since it allows testing for anti-Ca on the same unfixed tissue sections that are already used for the detection of most previously described anti-cerebellar antibodies by indirect immunofluorescence.

### Evidence for additional serum antibodies

While serum showed additional reactivity to capillaries in the ML and the WM, no such binding was found with CSF (Figure 2). The endothelial signal but not the PC staining disappeared after preadsorption with guinea pig liver powder (not shown). In addition, a fine punctate staining in the nuclei (nuclear dots) was found only with serum but not with CSF (Figure 10). However, no such fluorescence was present when the serum samples were tested at higher dilution, though the PC-specific fluorescence was still clearly detectable. Double labeling experiments identified the nuclear dot pattern found on brain cells as p80-coilin antibodies (Figure 10). Binding of the patient's serum to human full length coilin protein was also found in the protein microarray assay (median fluorescence units [FU] at 635 nm, 15676; median fluorescence of all proteins, 181; F-score, 10.62703; F-score cut-off, 3). Furthermore, serum antibodies to SS-A/Ro52 were detected, though no clinical signs of connective tissue disorders, such as Sjoegren syndrome or systemic lupus erythematodes, or of myositis were present in the patient, and no further extractable nuclear antigens such as nRNP/Sm, Sm, and SS-A/Ro-60, SS-B, Scl-70, PM-Scl, Jo-1, PCNA, double stranded DNA (dsDNA), centromer protein B (CEPB), nucleosomes, histones, and ribosomale P proteins were detectable.

### No evidence of previously described CNS autoantibodies

**Figure 11:**
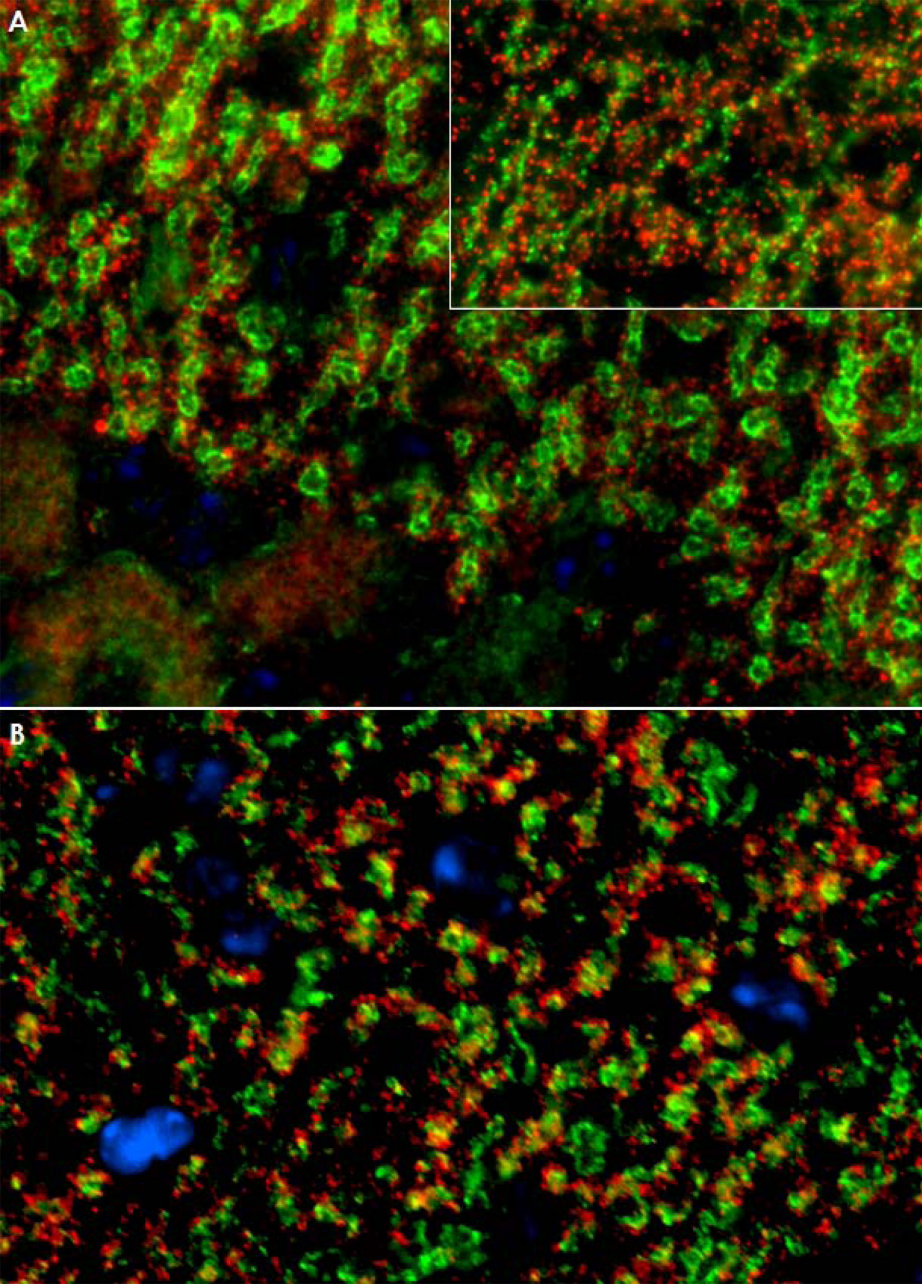
**No evidence for antibodies to Homer3 (A) or the metabotropic glutamate receptor 1 alpha (mGluR1α) (B) in the patient's CSF as demonstrated by double staining with commercial antibodies to these antigens**. While the patient's CSF preferentially stained Purkinje cell dendrites on mouse (Panel A and B) and monkey (Panel A, inset) tissue, Homer3 and mGluR1a immunoreactivity was widely restricted to dendritic spines. IgG antibodies to Homer3 and mGluR1α are depicted in red (AF568); the patient's IgG is labeled in green; yellow color indicates overlay of the two antibodies. Nuclei are shown in blue (DAPI).

**Figure 12:**
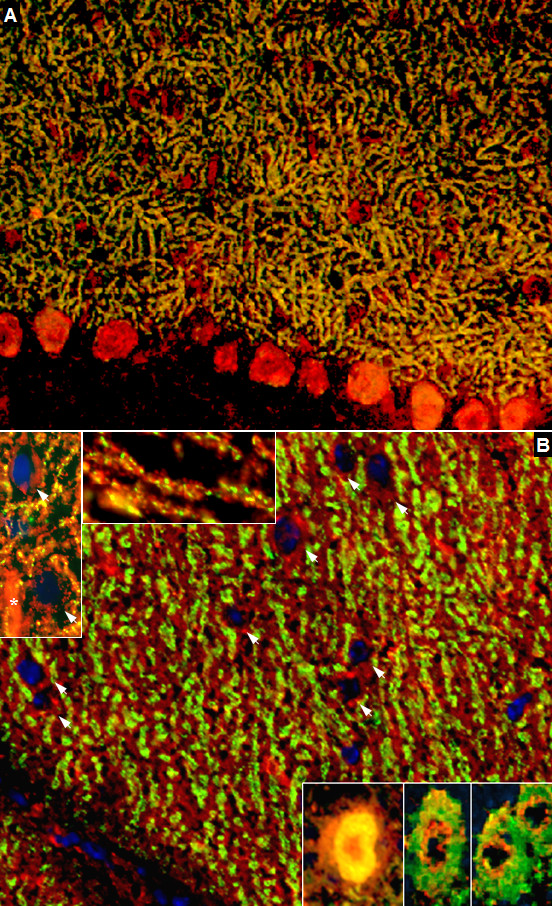
**No evidence for antibodies to the glutamate receptor delta 2 (GluRδ2) (A) or the protein kinase C type gamma (PKCγ) (B) in the patient's CSF as demonstrated by double staining with commercial antibodies to these antigens**. Note that the anti-GluRδ2 antibody as well as the anti-PKCγ antibody stained interneurons on mouse (A and B; *arrows*) and primate (B, left upper inset; *arrows*) cerebellar tissue, which were spared by the patient's antibody (cf. Figure 5). The anti-PKCγ antibody also bound to dendritic spines on monkey tissue that were not stained by the patient's antibody (B, right upper inset). In contrast to the patient's CSF, anti-PKCγ stained capillaries in primate brain (*asterisk*). Moreover, the cytoplasmic staining found with the anti-PKCγ antibody exceeded that caused by the patient's antibody and appeared to include the nuclear membrane (B, lower inset; left and right images represent different exposure times). IgG antibodies to GluRδ2 and PKCγ are depicted in red (AF568); the patient's IgG is labeled in green; yellow color indicates overlay of the two antibodies. Nuclei are shown in blue (DAPI).

Using two independent commercial line blot assays and a cerebellum western blot assay for the detection of classical paraneoplastic antibodies, no evidence was found for anti-Hu, anti-Ri, anti-Yo, anti-Ma, anti-Ta, anti-CV2/CRMP5, or anti-amphiphysin. Moreover, the PC-specific staining pattern found in our study did not correspond to the typical fluorescence patterns of any of those antibodies as described in the previous literature, or to that of ANNA-3[13], PCA-2[12], or anti-Tr[7,25]. Antibodies to Homer3[20], PKCγ [9], mGluR1[11,26], and GluRδ2[18,19], which were described in occasional patients with subacute cerebellar degeneration, and which are known to bind to PC somata and/or dendrites, and antibodies to GluR3 were ruled out by western blot analysis (Figure 7) and by double labeling experiments employing mouse and rat cerebellum tissue sections (Figure 11,12). Antibodies to VGCC, which are a rare cause of ataxia, were excluded in a commercial radioimmunoprecipitation assay (Euroimmun). Antibodies to NMDA receptors (subunit NR1a and NR2b) and AMPA receptors (subunits GluR1 and GluR2) were ruled out by indirect immunofluorescence on primate hippocampus tissue sections and by use of a cell-based assay employing HEK cells transfected with the respective antigens (Euroimmun). Commercial immunoblot assays revealed no evidence for ganglioside antibodies to GM1, GM2, GM3, GD1a, GD1b, GT1b, or GQ1b; antibodies against deaminated gliadin and antibodies to tissue transglutaminase were negative as well. Homer-3, PKCγ, Zic4, GAD, amphiphysin, and GluR2 were included also in the protein microarray, but were not recognized by the patient's IgG.

### Co-localization with the inositol-3-phosphate receptor type I

**Figure 13:**
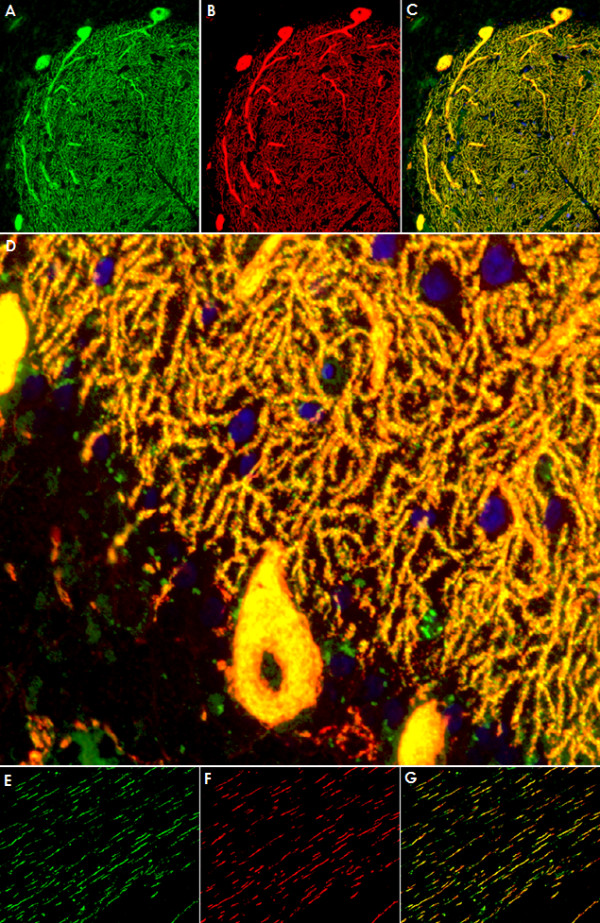
**Double labeling of a primate cerebellum tissue section with a commercial antibody to inositol-triphosphate receptor I (IP3RI), depicted in red (AF568), and the patient's CSF, shown in green (AF488)**. Nuclei are shown in blue (DAPI). An almost perfect overlay (yellow) of the two antibodies was found in the molecular layer and the Purkinje cell (PC) layer (Panels A-C), indicating that the patient's antibody targets an antigen expressed in close spatial proximity of IP3RI. Higher magnification (40×; D) suggests that the two antibodies might also bind to dendritic spines of PCs, though not all spines were stained by both antibodies. A fair, though less impressive, overlay was found in the white matter, where the two antibodies stained the same axons (E-G).

On cerebellum sections, we found an almost perfect overlay of the staining pattern found with the patient antibody in the molecular layer and that found with a commercial antibody to the inositol-3-phosphate receptor type I (IPR3I) (Figure 13A-C and 13D), which was used as a well established marker of PCs. However, an only partial overlay was found in the white matter (Figure 13E-F), indicating that the patient antibody targets an antigen that is spatially closely associated with IP3RI in some parts of the cerebellum but not identical to IP3RI. Accordingly, no band corresponding to the molecular weight of IP3RI (~300 kDa) was found by western blotting. Moreover, dot blot assays with three different IP3RI peptides (Santa Cruz) and a partial recombinant IP3RI protein (Abnova) were negative (not shown).

### Identification of ARHGAP26 as the target antigen

**Figure 14:**
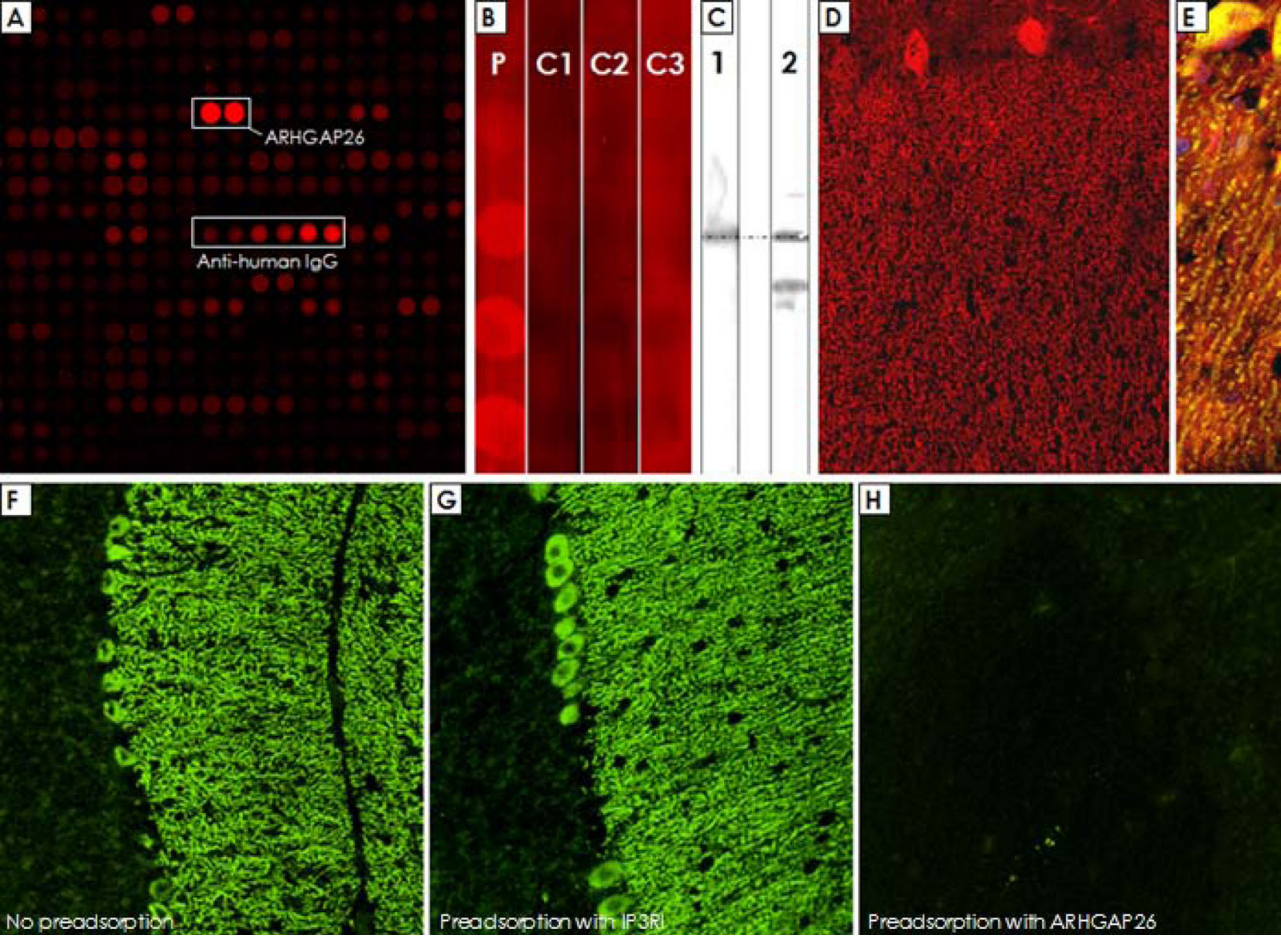
**Probing of a commercial protein microarray revealed strong binding of the patient's sera to human ARHGAP26 (Panel A)**. In accordance with this finding, binding of IgG from the patient's serum (Lane P), but not from three healthy controls (Lane C1-C3), to a recombinant human full length ARHGAP26 protein was found (Panel B). Western blotting confirmed the presence of ARHGAP26 in primate cerebellar extract (Panel C, lane 2), and incubation with the patient's CSF resulted in a band running at the same height as the ARHGAP26 band (Panel C, lane 1); the significance of the additional band recognized by the commercial antibody is unknown. The commercial ARHGAP26 antibody bound to the Purkinje cell layer and the molecular layer (Panel D) and showed a good overlay with the patient's IgG depicted in yellow (Panel E). Finally, preadsorption of the patient's CSF with the ARHGAP26 protein (Panel H), but not preadsorption with a control protein (Panel G), resulted in complete loss of binding to cerebellar tissue sections in an indirect immunofluorescence assay; panel F shows binding of IgG from a non-preadsorbed aliquot of the same CSF sample (exposure time was 2 sec in all cases to detect also low fluorescence signals).

Probing of a commercial protein microarray containing >9000 human full-length proteins revealed strong binding of the patient's serum to Rho GTPase activating protein 26 (ARHGAP26; alternative designations include GTPase regulator associated with focal adhesion kinase pp125, GRAF, and oligophrenin-1-like protein) (median fluorescence units [FU] at 635 nm, 55323; median FU of all proteins, 181; F-score, 38.7627; F-score cut-off, 3) (Figure 14A). In line with this finding, IgG from the patient's serum (lane 1; increasing protein dilutions from bottom of top) but not from healthy controls (lane 2-4) bound also to commercially available recombinant human full length ARHGAP26 from another manufacturer in a dot blot assay (Figure 14B). Accordingly, probing of a western blot of primate cerebellum extract with the patient's CSF revealed binding of IgG to a band running at the same height as a band detected with a commercial antibody to ARHGAP26 (Figure 14C). The same commercial ARHGAP26 antibody bound to the Purkinje cell layer and the molecular layer in an immunofluorescence assay and displayed overlay with the patient's IgG (Figure 14D-E). Finally, preadsorption of the patient's CSF with human ARHGAP26 protein but not preadsorption with control proteins resulted in complete loss of binding to cerebellum tissue sections (Figure 14F-H).

## Discussion

We identified a new serum and CSF autoantibody to Purkinje cells (PCs) in a patient with subacute cerebellar ataxia. Further experiments revealed ARHGAP26 as its target antigen. Our findings expand the panel of diagnostic serum markers of autoimmune ataxia and suggest a role of ARHGAP26 autoimmunity in the pathogenesis of this condition.

Autoantibody-associated subacute cerebellar ataxia is frequently of paraneoplastic nature[1,2]. Despite broad diagnostics, including repeat FDG-PET-CT scans, no tumor has been found in our patient 17 months after onset. However, paraneoplastic antibodies and the associated syndromes can precede tumor diagnosis by several years. In a large study on patients with anti-Yo antibodies, the most common paraneoplastic serum reactivity associated with autoimmune cerebellar ataxia, the neurologic syndrome preceded the diagnosis of cancer by up to 15 months and in many led to that diagnosis[4]. We can therefore not yet completely exclude a paraneoplastic origin of the syndrome.

Ataxia developed two weeks after a common cold in our patient. The close temporal relationship could indicate a causal link between the two events. Molecular mimicry has indeed been discussed to play a role in the pathogenesis of other autoimmune disorders of the nervous system such as the Guillain Barré syndrome[27]. Alternatively, infections can operate as a disease trigger in autoimmune disease[28]. Viral infections are well known to prompt disease activity in MS, and antecedent signs of viral infections were reported in 15-35% of neuromyelitis optica cases[29,30]. However, given the high prevalence of viral infections, the two events might well be causally unrelated.

The antibody bound almost exclusively to PCs. In good agreement with this finding, MRI and PET-CT did not display any sites of inflammation outside the cerebellum. However, it is of notice that our patient developed an increased startle response and a brisk head retraction reflex two months after onset, suggestive of symptomatic hyperekplexia, a condition mainly found in patients with brain stem disease[31]. Moreover, severe depression, restlessness, and anxiety occurred in our patient, which could indicate an involvement of the limbic system. We can therefore not completely rule out that other areas of the CNS were affected as well. On the other hand, rare cases of secondary hyperekplexia due to cerebellar pathology have indeed been reported in the literature[32], and a combination of steroid-induced and reactive depression sufficiently explains the patient's psychiatric symptoms.

There is some evidence for a direct role of the antibody in the pathogenesis of the condition. First, the antibody is highly specific for PCs. PCs are GABAergic neurons located in the cerebellar cortex and constitute the only output of motor coordination from the cerebellar cortex. Secondly, the antibody is produced intrathecally and at high titers. We found an extraordinarily high antibody index (AI) at onset (Table 1), strongly indicating that it is produced by clonally expanded B cells within the CNS[21]. Thirdly, it belongs to the IgG1 subclass and is capable of initiating complement C3b deposition in vitro, suggesting that it may act on PCs via complement-dependent mechanisms. Antibody-induced complement-mediated cytotoxicity is a well established feature in other autoantibody-associated disorders with presumed humoral pathogenesis such as neuromyelitis optica[33,34], though other direct effects such as antibody-dependent cell-mediated cytotoxicity or induction of apoptosis might have played a role as well. Finally, plasma exchange (PEx) and immunoadsorption (IA) was followed by clinical stabilization and moderate improvement. Unfortunately, no CSF or serum samples obtained shortly after PEx/IA were available for analysis. Testing of a paired CSF and serum sample taken after treatment with intravenous methylprednisolone and IVIG showed a decline of the Purkinje cell antibody index (AI) from 68 at month 1 to 12 at month 6, and normalization of the cell count and the blood brain barrier function. Interestingly, however, the decline of the AI value was mainly due to a decrease of the CSF titer from 1:2000 to 1:200, while the serum titer of the antibody did not change significantly.

However, passive transfer experiments, which alone could prove a direct pathogenic effect of the antibody, have not yet been performed. We can therefore not fully exclude that the antibody represents only an epiphenomenon, similar to the situation in many paraneoplastic neurological diseases, while the actual tissue damage is caused by other components of the immune system such as T cells. Indeed, the antibody targets a protein that resides at the inner side of the plasma membrane and in the cytoplasm. This is important since some authors believe that intracellular antigens might not be accessible to antibodies in vivo. Most neurological autoantibodies of proven pathogenic impact, such as antibodies to AQP4 in neuromyelitis optica [34-36], acetylcholine receptor in myasthenia gravis, VGCC in Lambert Eaton syndrome[37], and mGluR1 in paraneoplastic cerebellar degeneration[10] in fact target transmembrane proteins. Moreover, passive transfer of antibodies to nuclear antigens such as anti-Yo [38-40] have not produced clinical disease in animal studies. Instead, T cell-mediated immune mechanisms directed against the target antigen of the accompanying antibody have been proposed to play a role in those disorders [41-44].

However, there is still little direct evidence for a major role of T cells. Moreover, there are conditions in which antibodies against intracellular antigens were indeed shown to be of pathogenic impact, as proven by passive transfer. Sommer et al. found dose-dependent stiffness with spasms resembling human stiff-person syndrome in rats after injection of human serum containing high titers of antibodies to amphiphysin, a protein associated with the cytoplasmic surface of synaptic vesicles[45]. Interestingly, both amphiphysin and ARHGAP26 are involved in endocytosis[46]. One of the main roles of amphiphysin is to recruit dynamin to sites of clathrin-mediated endocytosis in GABAergic neurons[47]. Dynamin, a protein with GTPase activity, is important also in the clathrin-independent endocytic pathway, which is regulated by ARHGAP26[46], and ARHGAP26 is a strong interactor of dynamin[46]. Both proteins, amphiphysin and ARHGAP26, contain a BAR domain and a SH3 domain, though cross-reactivity of the patient's antibody with amphiphysin was excluded in our study by three independent methods (protein microarray; commercial line blot; and IHC). The patient's IgG indeed precipitated ARHGAP26 and co-precipitated dynamin from a mouse cerebellar extract as demonstrated by mass spectroscopy (data not shown), which is in perfect accordance with a recent study reporting co-precipitation of dynamin from rat brain cytosol by a non-human, ARHGAP26-specific antibody[46]. Besides anti-amphiphysin, a direct pathogenic effect of antibodies on intracellular proteins has also been shown for recoverin, which is located inside of retinal cells[48]. Anti-recoverin antibodies were demonstrated to enter retinal cells actively, probably by endocytosis, and uptake of anti-recoverin (but not of control IgG) induced caspase-dependent apoptosis[48]. Endocytotic uptake of antibodies has also been shown for a subset of anti-DNA antibodies [49-51]. Finally, a recent study demonstrated that PCs incorporate immunoglobulins of both the IgG and the IgM classes in vitro, independent of the immunoglobulin's reactivity with Purkinje cell surface antigens, giving raise to speculation as to whether paraneoplastic or other autoantibodies reactive with cytoplasmic or nuclear Purkinje cells antigens might be taken up intracellularly and could potentially produce cell injury and death[52].

ARHGAP26 has not previously been described as an autoimmune target in human or animal disease. However, mutations of ARHGAP26 are a rare cause of juvenile myelomonocytic leukemia[53], one of the most common pediatric myelodysplastic syndromes.

Further research is now needed to evaluate the exact role of anti-ARHGAP26 antibodies in the pathogenesis of cerebellar ataxia, involving the development of a passive transfer animal model. Moreover, diagnostic tests are to be developed to assess the frequency of anti-ARHGAP26 antibodies in patients with subacute ataxia.

## Conclusion

We describe a new autoantibody to Purkinje cell somata, dendrites and axons associated with subacute cerebellitis. The antibody targets ARHGAP26 and is produced intrathecally. Our findings indicate a role of autoimmunity to ARHGAP26 in the pathogenesis of subacute inflammatory cerebellar ataxia and expand the panel of diagnostic markers for this devastating condition.

## Competing interests

The authors declare that they have no competing interests.

## Authors' contributions

SJ conceived and designed the study. SJ, KPW, SH and HH performed and analyzed the experiments. SJ and BW drafted the manuscript. All authors participated in the critical revision of the manuscript; and all authors have given final approval of the version to be published.
